# Haemoparasites—Challenging and Wasting Infections in Small Ruminants: A Review

**DOI:** 10.3390/ani10112179

**Published:** 2020-11-22

**Authors:** Snorre Stuen

**Affiliations:** Department of Production Animal Clinical Sciences, Norwegian University of Life Sciences, N-4325 Sandnes, Norway; snorre.stuen@nmbu.no

**Keywords:** haemoparasites, tsetse flies, ticks, goat, sheep, wasting disease

## Abstract

**Simple Summary:**

Vector-borne haemoparasites in small ruminants are widespread, although only scattered information is available on their occurrence. Haemoparasites occur in the genera *Anaplasma*, *Babesia*, *Ehrlichia*, *Mycoplasma*, *Theileria* and *Trypanosoma*, of which several species cause challenging and wasting conditions with severe impact on the small ruminant industry. However, the controls of these infections are challenging tasks, especially since several of the available medical drugs have negative environmental consequences. In addition, vectors may spread across the world to new geographical areas, especially related to climate change and increased globalization. These changes may have a profound impact on infection ecology and disease management. Integrated control strategies should be implemented, such as breed resistance against vectors and infections, and strategic vector and infection control.

**Abstract:**

Haemoparasites include bacteria, mycoplasma, protozoa and flagellates inhabiting the bloodstream of living hosts. These infections occur worldwide and are transmitted by vectors, especially ticks and tsetse flies. Geographical distribution varies due to movements of animals and vectors between geographical areas, and even between countries and continents. These changes may be caused by climate change, directly and indirectly, and have a huge effect on the epidemiology of these microbes. Active and ongoing surveillance is necessary to obtain reliable maps concerning the distribution of these infections in order to do proper risk assessment and efficient prophylactic treatment. Genera *Anaplasma*, *Ehrlichia*, *Mycoplasma*, *Babesia*, *Theileria* and *Trypanosoma* include common haemoparasite species in small ruminants causing a variety of clinical manifestations from high fatality rates to more subclinical infections, depending on the species or strain involved. These infections may also cause ill-thift or long-lasting wasting conditions. Life-long infections are a common feature of these pathogens. The present review will focus on haemoparasites in small ruminants, especially related to challenging and wasting infections.

## 1. Introduction

In recent years, a series of vector-borne diseases have spread to new geographical areas across the world, of which several are caused by haemoparasites. Haemoparasites are pathogens that inhabit the bloodstream of the host and includes microorganisms such as bacteria, mycoplasma, protozoa and flagellates. These infections occur on all continents, whereas the distribution of these infections changes continuously due to the migration and transportation of vectors and animals and an increased globalization of both live animals and their products. Short- and long-distance movements of vectors and hosts are driven directly or indirectly by climatic changes. These changes will have a huge effect on the distribution and establishment of both pathogens and vectors [1,2]. Climate change may provide new habitats for living organisms and cause a climatic-driven behaviour adaptation that may ease the spread of infections due to an increase in the vector–pathogen–host interactions [3]. Successful colonization of vectors in new areas will, however, depend on both available hosts and suitable habitats.

Climate-warming models predict that several tick species are likely to establish more northern permanent populations [4]. In addition, millions of ticks are annually spread by migrating birds, making the possibility for ticks and pathogens to be transmitted and established in new areas [5]. Other arthropod vectors, such as *Glossina* species, may also spread from sub-Saharan Africa, which is their normal distribution area. Although, a further rise in temperature in the current areas of tsetse flies may increase larvae mortality and thereafter reduce the population of tsetse flies [3]. However, limited data are available concerning the effect of global warming on the local distribution of these flies. 

Around 900 species of ticks have been described [6]. Ticks in the genera *Amblyomma*, *Haemophysalis*, *Hyalomma* and *Rhipicephalus* are frequently associated with small ruminants, but *Dermacentor* and *Ixodes* ticks are also important vectors [7]. In Europe, at least 12 species have been found on small ruminants, whereas the main species are *Dermacentor marginatus*, *Haemaphysalis punctata*, *Ixodes ricinus* and *Rhipicephalus bursa* [8]. Several ticks may infest small ruminants on other continents; however, only scattered information on their distribution is available. 

Tsetse flies (*Glossina* spp.) are important vectors of trypanosomes in sub-Saharan Africa. More than 31 species or subspecies of tsetse flies have been identified, whereas 8–10 species are considered to be of economic importance [9]. However, other vectors of trypanosomes, such as biting flies in the genera *Tabanus* and *Stomoxys* are important mechanical vectors in areas without *Glossina* spp, such as in South Africa and Asia [10]. 

Haemoparasites may cause acute disease with variable clinical symptoms depending on the species or strains of the pathogen involved. Persistent infection is a common feature, while chronic and wasting infections are especially related to some species (Table 1). Long-lasting infections may often lead to wasting conditions. The present review will focus on haemoparasites in small ruminants related to these wasting diseases. 

## 2. Mycoplasma ovis

### 2.1. Species and Distribution

*Mycoplasma ovis* (formerly classified as *Eperythrozoon ovis*) is an uncultivated pleiotropic bacterium which parasitizes erythrocytes [11]. In the last decade, a similar organism, *Candidatus* Mycoplasma hemovis, has been detected. However, recent investigation suggests that these two species may represent the same organism [12]. 

*M. ovis* occurs worldwide in small ruminants, although there is a lack of information concerning the actual distribution, especially in goats [11,13]. 

### 2.2. Transmission and Reservoir 

Transmission of *M. ovis* seems to occur mainly by biting insects (flies, lice, keds and mosquitos), but also by needles or surgical instruments. Ticks may occasionally play a role in pathogen transmission [11]. In addition, oral transmission has been shown to occur—whereas only one infected erythrocyte seems to be enough to transmit the infection [14]. No indication of vertical transmission has so far been reported. 

Natural reservoirs besides sheep and goats are unknown; however, cervids seem to be infected with *M. ovis* [15,16]. In recent years, human infections have been reported [17,18]. 

### 2.3. Clinics and Diagnosis 

*Mycoplasma ovis* causes haemolytic anaemia in small ruminants. Clinical symptoms also include hemoglobinuria, unthriftiness, stiffness and reduced weight gain [14,19]. Fever and icterus are not regularly observed. However, subclinical or latent infections seems to be the rule. Clinical disease is often triggered by other factors such as malnutrition or gastrointestinal parasitism. Susceptibility to the infection seems to vary between sheep and goats [20], of which clinical disease is rarely observed in goats [13]. In sheep, however, chronic infection may occur, an ill-thrift condition in lambs where they become small, pot-bellied, emaciated and often with a fatal outcome [14,21]. *M. ovis* may lead to severe livestock losses mainly due to retardment of weight gain in lambs and loss of condition in mature sheep [22]. 

The organism can be found on erythrocytes by light microscopy with the use of stained blood smears [23]. Up to 100% of the erythrocytes can be infected, although normally only a few percentages are detected by microscopy. Low infection level may be difficult to diagnose by direct microscopy and require examination of repeated blood smears. Serology can be used however molecular methods are often necessary in order to verify the diagnosis [11]. Co-infections with other pathogens (such as *Anaplasma ovis* and *A. phagocytophilum*) may occur, aggravating the clinics and challenging the diagnostics [17]. 

### 2.4. Treatment and Control 

Neoarsphenamine, antimosan or oxytetracycline have been recommended for sheep. No specific treatment evaluations have been reported in goats [13]. Treatment may diminish clinical symptoms but may not clear the organisms from the body. Controls involve good preventive measures such as enough nourishment of good quality, minimizing the risk of biting flies and avoiding the spread of the organism by needles and surgical instruments. 

## 3. *Babesia* spp. 

### 3.1. Species and Distribution 

Babesiosis is caused by protozoa in the genus *Babesia*, of which the main species affecting small ruminants are *B. ovis*, *B. motasi* and *B. crassa. B. ovis* is the most pathogen species and an important tick-borne haemoparasite in sheep worldwide [24], while *B. motasi* is the dominant species in goats [13]. *B. motasi* may consist of at least two species/subspecies, which may differ in pathogenicity [24,25]. The situation regarding *Babesia* spp. of small ruminants is, however, rather confusing and includes several new and unclassified strains/species [25,26]. 

*B. ovis* has a worldwide distribution, while *B. motasi* has been detected in Europe, central and northern Africa, Asia and Central America [24,27] *B. crassa* has only been detected in a few countries, including Iran and Turkey, and is known only in areas were *Hyalomma* and *Rhipicephalus* ticks are common [13]. 

### 3.2. Transmission and Reservoir 

*Rhipicephalus* ticks, such as *R. bursa*, *R. sanguineus* and *R. turanicus* have been implemented in the transmission of *B. ovis*, whereas *R. bursa* is the only vector so far that is reported to transmit *B. ovis* transovarially [28]. Other tick species may occasionally be involved in the transmission cycle [29,30]. In contrast, *B. motasi* is mainly associated with *Haemophysalis punctata* or other ticks in the genus *Haemophysalis* [13]. Sheep and goats are the only known vertebrate reservoir hosts of *B. ovis* and *B. motasi.* In addition, ticks may serve as natural reservoirs [31]. *B. ovis* is known to be maintained in several generations of ticks without reinfections [32]. 

### 3.3. Clinics and Diagnosis 

Acute babesiosis caused by *B. ovis* is characterized by apathy, fever, anaemia, jaundice and haemoglobinuria, and mortality may occur. The infection varies in the degree of severity associated with age, immunity and health status. Young animals seem to develop less clinical symptoms due to innate immunity. Infection is normally mild in indigenous sheep, whereas severe signs are often related to animals introduced from non-endemic areas. In goats, natural infection with *B. ovis* has only been reported in few areas and only as a subclinical condition [13,33]. Animals that survive the acute infection become low-level carriers and could be persistently infected for years without apparent clinical signs [34]. However, relapses may occur under stressful conditions, such as poor nutrition and other infections [31].

*B. motasi* produces in general a mild clinical response characterized by fever, haemoglobinuria and anaemia, but is rarely responsible for significant death losses [30]. However, more severe symptoms have been recorded in goats with high morbidity and mortality. Death may occur 48 h after the onset of clinical symptoms. Chronic and wasting condition may occur with anaemia and ill thrift [13]. In contrast, *B. crassa* infection in small ruminants seems to be non-pathogenic [34].

Diagnosis is based on the clinical symptoms mentioned above and the demonstration of typical protozoa in stained blood smears. However, the infection rate rarely exceeds a few percentages during the acute phase of the infection and may be difficult to detect by blood smear microscopy [30,31]. However, PCR and serological tests are available, especially PCR-analysis should be used to detect a subclinical *B. ovis* infection [27,28]. The most characteristic findings at post-mortem are anaemia, icterus and haemoglobinuria. 

### 3.4. Treatment and Control 

Earlier results indicate that treatment of babesiosis (*B. ovis* infection) with imidocarb diproprionate was efficient [35]. In addition, diminazene aceturate may be efficient against *B. ovis* The development of new drugs is in progress [36]. In goats, diminazene has been used against *B. motasi* [13]. Tick control is important in order to reduce the occurrence of babesiosis (see below). 

## 4. *Theileria* spp. 

### 4.1. Species and Distribution 

Theileriosis is caused by parasitic protozoan that belongs to the genus *Theileria*. In small ruminants, *Theileria* genotypes comprise a heterologous group and consists of several species such as *T. lestoquardi* (formerly *T. hirci*), *T. ovis*, *T. recondita*, *T. annulata*, *T. uilenbergi*, *T. separata*, *T. luwenshuni*/sp. OT1 and *Theileria* sp. OT3 [30,37,38,39]. In addition, several new and uncharacterized isolates of *Theileria* of small ruminants have been reported [39,40]. 

*Theileria* species in small ruminants are widespread, particularly in tropical and subtropical regions of Africa, the Middle East, eastern and southern Europe and Asia [41], although only scattered information is available. 

### 4.2. Transmission and Reservoir 

A variety of tick vectors are involved, mainly in the genera *Haemaphysalis*, *Hyalomma* and *Rhipicephalus* [42] (Table 1). For instance, *T. lestoquardi* is mainly transmitted by *Hyalomma anatolicum* and *Haemophysalis qinghaiensis*, and *T. ovis* by *R. bursa* and *H. punctata.* Transplacental transmission of *T. lestoquardi* has been reported in both sheep and goats [43,44]. In addition, *T. luwenshuni* and *T. uilenbergi* seem to be related to *H. qinghaiensis* and *H. longicornis* [39]. Limited data are available concerning natural hosts of the different *Theileria* species. *T. lestoquardi* and *T. ovis* have so far only been reported from sheep and goats, while *T. uilenbergi* and *T. luwenshuni* have also been detected from sika and red deer [42].

### 4.3. Clinics and Diagnosis 

Only *T. lestoquardi*, *T. luwenshuni* and *T. uilenbergi* are considered pathogenic for small ruminants, of which *T. lestoquardi* is the most pathogenic species [40,45]. Susceptibility to the infection varies due to internal (genetics, breed, lactation, parturition) and external (nutrition, concomitant infections) factors. In addition, the infection dose may influence the outcome of the infection, whether it will become subclinical or lethal [37].

*T. lestoquardi*, causing malign theileriosis, may introduce severe infection in sheep with high mortality rates (46–100%) [30,46]. In malign theileriosis, an acute form is common, but subacute and chronic forms may also occur. Clinical signs in the acute form are fever, cessation of rumination, swelling of superficial lymph nodes, diarrhoea, jaundice and haemorrhages (submucosal and subcutaneous). In chronic infections, intermittent fever, inappetence, anaemia, jaundice and emaciation are recorded. In general, *Theileria* spp. seems to cause much less symptoms in goats than in sheep [13]. 

Other *Theileria* species may cause mild or even inappreciable clinical symptoms, such as *T. ovis* causing benign theileriosis [24]. However, benign *Theileria* species may also cause significant production losses in imported, immunocompromised or stressed animals [37].

Diagnosis is based on the detection of protozoa in stained blood smears, lymph node biopsies (live animals) or in lymph nodes or spleen smears at post-mortem [30]. However, parasitaemia fluctuates and may drop below detection level, so microscopic detection of parasites can be difficult and does not readily allow for differentiation between species [47]. Serology are useful for identifying infected animals, although not specific to identify species. Detection of *Theileria* in the blood should be performed by molecular methods, such as reverse line blot and real-time PCR [48,49]. Post-mortem analysis includes anaemia, icterus, intramuscular and subcutaneous oedema, splenomegaly and hepatomegaly [37]. 

### 4.4. Treatment and Control 

Specific treatment of caprine theileriosis is lacking. For *T. lestoquardi*, a single injection of parvaquone or buparvaquone given at two occasions may be used. A single dose of halofuginone is also reported to be efficient [18,30]. *Theileria* species causing mild or even inappreciable clinical symptoms is usually not treated. A vaccine is not commercially available. Tick control measures should be considered for controlling the disease and in order to limit the distribution and expansion of the pathogenic species. However, several of these treatments are not environmentally friendly and may also cause chemical residues in animal products. Control should be based on an integrated control against both parasites and vectors.

## 5. Trypanosoma spp.

### 5.1. Species and Distribution 

Trypanosomiasis is caused by haemoflagellates in the genus *Trypanosoma*. Several species are involved although not all of them seem to be pathogenic for small ruminants. Animals are often infected with more than one species or strain [50]. The most important species for small ruminants are *T. congolense*, *T. vivax*, *T. brucei* and *T. evansi* [51]. However, recent molecular methods indicate that *T. evansi* may is a subspecies or strain of *T. brucei* [52]. In addition, several strains of trypanosomes occur within each species [13].

As already mentioned, the most important species for small ruminants are *T. congolense*, *T. vivax*, *T. brucei* and *T. evansi* [10]. However, their real distribution is unknown. The infection is widespread in tropical and subtropical areas, especially in sub-Saharan Africa, but *Trypanosoma* species have also been detected in Europe, West Indies, Central and South America and Asia [13,30]. Outside Africa, *T. evansi* have been established in Asia, while both *T. vivax* and *T. evansi* have been established in South America [10,30].

### 5.2. Transmission and Reservoir 

In Africa, most cases seem to be transmitted by tsetse flies (*Glossina* spp.), mainly in sub-Saharan Africa. However, the geographical distribution of trypanosomes extends beyond the tsetse-infested areas, which may be due to animal movement and mechanical transmission by other vectors. *T. evansi* and *T. vivax* have, for instance, been shown to be mechanically transmitted by blood-sucking insects (especially in the *Stomoxydae* and *Tabanidae* families) in South America and Asia [10,30]. However, cyclic transmission in the vector seems only to involve *Glossina* species. In addition, *Trypanosma* spp. may be transmitted by contaminated needles during medication and vaccination, which seems to be one reason for the widespread distribution in tsetse-free areas [53]. Transplacental transmission of *T. evansi* and *T. vivax* in sheep has also been reported [53,54].

Concerning the animal reservoir, little is known about the natural animal reservoir. Trypamosomes usually affect several animal species. Infected sheep and goats are carriers and can be the main source for maintenance of the infection [55]. Small ruminants may also function as a reservoir of trypanosomes affecting other mammalian species including humans [56]. 

### 5.3. Clinics and Diagnosis 

The main clinical symptom related to trypanosomiasis is anaemia. Infected animals may show intermitting fever, decreased appetite, anorexia, rumen atony, enlarged lymph nodes and progressive loss of condition. Jaunice and hemoglobinuria are uncommon findings. Reduced milk production, reduced fertility and increased mortality may also be an indication of trypanosomiasis [10]. Clinical signs depend on the species of *Trypanosoma* involved. *T. vivax* and *T. congolense* cause acute, subacute and chronic disease, in which the acute form may cause death or recovery within four to six weeks. In chronic cases, severe emaciation may occur. *T. congolense* infection often results in cyclic fever and parasitaemia, and the mortality rate is high if the infection has lasted for more than 12 weeks. In *T. brucei* cases, CNS-symptoms such as head pressing, circling and opisthotonus may occur, often with a fatal outcome [13]. 

Goats with chronic trypanosomiasis may be more susceptible to helminthiasis probably as a result of immunosuppression. A reduction in milk quality and production have been observed in *T. vivax*-infected goats [57]. In addition, trypanosome infection in goats may be associated with ovarian dysfunction, irregular estrus cycles and testicular atrophy [13]. 

Variation of the clinical presentation is caused by several factors, involving strains/species of trypanosomes and genetic variation within and between breeds. It is important to notice that chronically infected animals can remain subclinical for a long period. However, reactivation of clinical symptoms may occur due to nutritional and physical stress or infection with other pathogens. Infection should therefore always be suspected in animals showing relapsing fever, progressive weight loss, abortion and especially anemia [55]. 

Anemia and emaciation in animals from areas with tsetse flies indicate trypanosomiasis. Diagnosis is based on organisms found in stained blood smears or tissues. However, trypanosomes may be difficult to identify in chronic infections due to low parasitaemia. To confirm *T. brucei* infection, examination of lymph node aspirate or mouse inoculation are the preferred methods. Several serological tests for antibody detection are available. In addition, PCR methods are developed, but demand well-equipped labs. Necropsy of acute cases will reveal anaemia, lymph node enlargement, splenomegaly, petechial bleedings (mucosa/serosa) and hydropericardium [13]. 

### 5.4. Treatment and Control 

Trypanosomiasis has for decades been a major concern in small ruminant production, especially in sub-Saharan Africa [13]. In the African countries, it has been estimated that around 260 million small ruminants have reduced productivity due to these haemoparasites [32]. 

Over the years, extensive efforts have been made to control and eliminate the tsetse flies, based on the ground spraying of insecticide, sterile insect release technique, bush clearing and destruction of fly habitat, use of traps and the use of insecticide-treated livestock [10]. However, these interventions have only partly been successful and are often associated with negative environmental consequences such as destruction of the insect fauna, pollution of water bodies and deforestation. In addition, residues may occur in animal products [58]. Curative and prophylactic use of trypanocidal drugs administrated by the farmers, remains the most important method of controlling animal trypanosomiasis [9]. A limited number of trypanocidal compounds are available, such as diminazene aceturate, isometamidium and homidium chloride. However, the use of a few therapeutic drugs with a limited efficacy have fueled the widespread emergence of resistance [58]. In addition, there are numerous constraints on the control of trypanomosiasis, such as reservoirs in the wild animal populations, lack of vector control and lack of economic resources [13]. 

The most important method in the future to control this infection may be breeding for trypanotolerance, since indigenous breeds seem to tolerate the infection and perform better than imported breeds or crossbreeds. Trypanotolerance offers farmers an important option for sustainable production in risk areas. Preliminary field studies indicate that sheep and goat breeds such as Dwarf West African goat, Small East African goat, Djallonke sheep and Red Masai sheep are trypanotolerant. In these cases, trypanotolerance seems to be more related to resilience than to resistance [59]. However, factors such as helminthiasis, inadequate nutrition level and the severity of the trypanosome infection involved (strain, dose) may have a negative effect on this tolerance [13,51]. 

In sub-Saharan Africa, the major fronts against trypanosomiasis are the reduction or elimination of the tsetse populations. No effective vaccine is yet available, due to challenges identifying proper vaccine candidates [13]. The lack of a suitable vaccine has facilitated an overuse of the few available drugs. In addition, ongoing efforts to control the tsetse flies have been largely ineffectual, partly due to political instabilities and armed conflicts in the actual areas [58]. Risk-based control and monitoring activities are the foundation to control disease outbreak of trypanosomiasis [10]. 

## 6. Haemoparasites Rarely Involved in Wasting Disease 

Haemoparasites in the genera *Anaplasma* and *Ehrlichia* are also important infections in the small ruminant industry, which seldom cause wasting conditions, except for tick pyaemia related to a primary *A. phagocytophilum* infection. The most common infections among these species will only briefly be mentioned. 

### 6.1. Anaplasma ovis 

*A. ovis* is a rickettsia with a widespread infection in small ruminants, especially in the Mediterranean countries and central Europe. *A. ovis* is spread by a variety of ticks, particularly *Rhipicephalus* and *Dermacentor* species. The infection in small ruminants may cause haemolytic anaemia, weight loss, abortion and even death [60]. However, subclinical infection is common, and an active surveillance is necessary in order to investigate the real distribution [61]. Outbreaks of severe illness in sheep and goats are rare and seem to occur mainly under stressful conditions. However, infection with *A. ovis* may predispose animals to other infections and parasite infestation resulting in clinical disease or even death and may therefore have an impact on health, milk and meat production in small ruminants [61]. Recently, outbreaks of anaplasmosis in lambs due to *A. ovis*, have been reported, including severe anaemia and icteric carcasses [62,63]. The pathogenesis of this strain has to be further unravelled.

### 6.2. Anaplasma phagocytophilum (formerly Ehrlichia phagocytophila) 

*A. phagocytophilum* causes tick-borne fever (TBF) in small ruminants [64]. The infection is mainly associated with *Ixodes* spp. ticks. *A. phagocytophilum* infection is widespread in Europe, while only scattered information is available from other continents. Only a few cases of *A. phagocytophilum* infection in goats have so far been reported.

TBF may cause severe economic and welfare challenges in the sheep industry. The most characteristic symptom of the disease in domestic ruminants is high fever. TBF is seldom fatal, unless complicated by other infections. The infection may cause immunosuppression in sheep that makes affected animals vulnerable to secondary infections, such as tick pyaemia (caused by *Staphylococcus* spp.) or *Bibersteinia*/*Mannheimia* septicaemia [64,65]. Other complications include abortion and reduced milk yield, impaired spermatogenesis in males, and reduced weight gain in young animals. Tick pyaemia, a wasting and crippling condition caused by *Staphylococcus* aureus, a common manifestation related to a primary *A. phagocytophilum* infection, is a severe challenge in some countries [64,66]. In contrast, secondary bacterial infections are not a significant part in the reported outbreaks of caprine TBF [18]. 

Several variants/strains of *A. phagocytophilum* have been characterized that seem to be involved in different natural enzootic cycles. Natural reservoirs of these variants are mostly unknown, although red deer seems to be involved as a reservoir host for strains causing TBF in sheep [64]. Current control strategies are based on the reduction of tick infestation by acaricides on tick pasture. This is mostly done be dipping or pour-on applications of pyrethroids [64]. 

### 6.3. Ehrlichia ruminantium (formerly Cowdria ruminantium) 

*E. ruminantium* causes heartwater, a serious condition in ruminants with high mortality. The infection is transmitted by ticks in the genus *Amblyomma*, especially *A. variegatum* and *A. hebraeum* [67]. Heartwater is an endemic disease in domestic and some wild ruminants throughout sub-Saharan Africa, including islands near eastern Africa, but occurs also in the Caribbean [68]. There are four clinical forms of heartwater—peracute, acute, subacute and subclinical—whereas the acute form is most common [18]. The disease is characterized by the sudden onset of high fever, nervous signs, and rapid and abdominal breathing. *E. ruminantium* may cause high mortalities (more than 90% is recorded in sheep) related to breed, age and bacterial strains [69,70]. Subclinical and mild cases are common in young animals and in local indigenous breeds. Chronic infection has not been reported. 

Although heartwater has been known for more than a century, it is still considered a major obstacle against the expansion and development of the livestock industry in southern Africa. The current methods for heartwater control include the use of acaricides to control the ticks, antibiotic prophylaxis, immunization by infection and treatment, farming with resistant breeds and establishment of endemic stability. Currently, there is no safe, user-friendly and reliable vaccine commercially available [71,72].

## Figures and Tables

**Table 1:** Occurrence of mortality and wasting condition among haemoparasites in small ruminants.

Species	Vector	Mortality	Wasting Condition
*Anaplasma ovis*	Ticks, especially *Rhipicephalus* spp. and *Dermacentor* spp.	Low	Uncommon
*A. phagocytophilum*	Ticks, especially *Ixodes* spp.	High ^a^–Low	Common ^a^
*Babesia ovis*	Ticks, especially *Rhipicephalus bursa*	High–Low	Uncommon
*B. motasi*	Ticks, especially *Haemophysalis punctata*	Low–High	Uncommon
*Babesia* spp.	Ticks	Low	Uncommon
*Ehrlichia ruminantium*	Ticks, especially *Amblyomma* spp.	High	Uncommon
*Mycoplasma ovis*	Mainly biting flies	Low	Uncommon
*Theileria lestoquardi*	Ticks, especially *Hyalomma anatolicum* and *Haemophysalis. qinghaiensis*	High	Common
*Theileria ovis*	Ticks, especially *R. bursa* and *H. punctata*	Low	Uncommon
*Theileria* spp.	Ticks	Low–High	Uncommon
*Trypanosoma congolense/brucei/evansi/vivax*	*Glossina* spp.—other biting flies	High–Low	Common
*Trypanosoma* spp.	*Glossina* spp.—other biting flies	Low	Uncommon

^a^ due to secondary infections.
